# Anticonvulsant Use in Older Age Bipolar Disorder in a Global Sample from the Global Aging and Geriatric Experiments in Bipolar Disorder Project: Utilisation d’anticonvulsivants pour le traitement des troubles bipolaires du sujet âgé auprès d’un échantillon mondial provenant du projet GAGE-BD

**DOI:** 10.1177/07067437251372190

**Published:** 2025-09-22

**Authors:** Katie C. Bodenstein, Myriam Lesage, Paola Lavin, Sigfried Schouws, Melis Orhan, Alexandra Beunders, Osvaldo P. Almeida, Kursat Altinbas, Vicent Balanzá-Martínez, Izabela G. Barbosa, Hilary P. Blumberg, Farren B.S. Briggs, Cynthia V. Calkin, Orestes V. Forlenza, Brent Forester, Ariel G. Gildengers, Benno C.M. Haarman, Tomas Hajek, Beny Lafer, Paula Nune, Benoit Mulsant, Andrew T. Olagunju, Regan E. Patrick, Joquim Radua, Kaylee Sarna, Christian Simhandl, Jair C. Soares, Ashley N. Sutherland, Nicole Fiorelli, Antonio L. Teixeira, Shangying Tsai, Eduard Vieta, Joy Yala, Lisa Eyler, Annemiek Dols, Martha Sajatovic, Soham Rej

**Affiliations:** 1 Department of Psychiatry, 5620 McGill University, Jewish General Hospital/Lady Davis Institute, Montreal, QC, Canada; 2 Department of Old Age Psychiatry and Neuropsychiatry, 36522 GGZ inGeest, Amsterdam, Netherlands; 3 Clinical Psychology Unit, 4496 Institute of Psychology, Leiden University, Leiden, The Netherlands; 4 Specialized Mental Health Care, 36522 GGZ inGeest, Amsterdam, The Netherlands; 5 3431 Institute for Health Research of the University of Notre Dame Australia & 172098 Medical School of the University of Western Australia, Perth, Australia; 6 Department of Psychiatry, 553310 Atlas University, Istanbul, Turkey; 7 Teaching Unit of Psychiatry and Psychological Medicine, Department of Medicine, 16781 University of Valencia, CIBERSAM, INCLIVA, Valencia, Spain; 8 Mental Health Department, Medicine School, Minas Gerais University, Belo Horizonte, Brazil; 9 Department of Psychiatry, 12228 Yale School of Medicine, New Haven, Connecticut, USA; 10 Department of Public Health Sciences, 12235 University of Miami Miller School of Medicine, Miami, Florida, USA; 11 Departments of Psychiatry and Medical neuroscience, 3688 Dalhousie University, Halifax, Canada; 12 Laboratory of Neuroscience (LIM-27), Department and Institute of Psychiatry, 117265 HCFMUSP, Faculdade de Medicina da Universidade de São Paulo, São Paulo, SP, Brazil; 13 Department of Psychiatry, 1867 Tufts Medical Center, Boston, USA; 14 Department of Psychiatry, 12317 University of Pittsburgh School of Medicine, Pittsburgh, Pennsylvania, USA; 15 Department of Psychiatry, 10173 University Medical Center Groningen, University of Groningen, Groningen, Netherlands; 16 Department of Psychiatry, 3688 Dalhousie University, Halifax, Canada; 17 Department of Psychiatry, University of São Paulo Medical School, São Paulo, Brazil; 18 Department of Psychiatry, 12366 University of Toronto, Center for Addiction & Mental Health, Toronto, Canada; 19 Department of Psychiatry and Behavioral Neurosciences, 62703 McMaster University/St Joseph's Healthcare Hamilton, Hamilton, Ontario, Canada; 20 Division of Geriatric Psychiatry, 24472 McLean Hospital, Belmont, MA, USA; 21 Harvard Medical School, Boston, MA, USA; 22 456032 IDIBAPS, CIBERSAM, Barcelona, Spain; 23 12304 Case Western Reserve University School of Medicine, Cleveland, Ohio, USA; 24 Medical Faculty, Bipolar Center Wiener Neustadt, Sigmund Freud University Vienna, Vienna, Austria; 25 Department of Psychiatry and Behavioral Sciences, 12339 University of Texas/McGovern Medical School, Houston, Texas, USA; 26 Department of Psychiatry, 8784 University of California San Diego, San Diego, CA, USA; 27 Desert-Pacific Mental Illness Research Education and Clinical Center, VA San Diego Healthcare System, San Diego, CA, USA; 28 Biggs Institute, 14742 University of Texas Health Science Center at San Antonio, San Antonio, Texas, USA; 29 Department of Psychiatry, School of Medicine, College of Medicine, 38032 Taipei Medical University, Taipei; 30 Bipolar and Depressive Disorders Unit, 16493 Hospital Clinic, University of Barcelona, IDIBAPS, CIBERSAM, ISCIII, Barcelona, Catalonia, Spain; 31 Department of Psychiatry, 8784 University of California, San Diego, La Jolla, USA; 32 Department of Psychiatry, 8124 UMC Utrecht Brain Center, University Medical Center Utrecht, Utrecht, The Netherlands; 33 Department of Psychiatry, Amsterdam UMC, Amsterdam Neuroscience, Amsterdam, The Netherlands; 34 University Hospitals Cleveland Medical Center, Cleveland, Ohio, USA; 35 School of Nursing and Midwifery, 1596 Queen's University Belfast, Belfast, UK

**Keywords:** older age bipolar disorder, anticonvulsants, geriatric

## Abstract

**Background:**

Anticonvulsants are an essential treatment for bipolar disorder; however, there is relatively little known about their use in older age bipolar disorder (OABD). In this paper, which leverages a large international dataset, we aim to 1) describe the use of anticonvulsants in OABD compared to younger age bipolar disorder (YABD; ages <50 years old) and 2) explore any demographic/clinical correlates.

**Methods:**

A secondary analysis was conducted on the international data from the Global Aging and Geriatric Experiments in Bipolar Disorder project. The main objective was to report the prevalence of anticonvulsant use in OABD over 50 years old (mean age = 62.27) and the most prescribed anticonvulsant. Additional analysis explored any demographic and clinical correlates associated with anticonvulsant use. Generalized linear mixed models were used for this analysis.

**Results:**

Of the 2,691 participants with bipolar disorder who had anticonvulsant prescribing data, 34.4% (n = 926) used anticonvulsants at the time of study. Rates of anticonvulsant prescribing did not significantly differ between OABD and YABD groups (36.7% (n = 666) vs. 29.7% (n = 260)). Anticonvulsant prescribing patterns for OABD and YABD did not significantly differ, with valproate as the most prescribed anticonvulsant. OABD anticonvulsant users had less lithium use, more antidepressant use, more rapid cycling, more mood episodes and more cardiovascular comorbidities compared to nonusers.

**Conclusion:**

Anticonvulsant use was similar in OABD and YABD. A number of important clinical correlates of anticonvulsant use were identified.

## Résumé

**Contexte:**

Les anticonvulsivants sont un traitement essentiel contre le trouble bipolaire, mais leur utilisation pour traiter les troubles bipolaires du sujet âgé est relativement peu connue. Dans cet article, lequel s’appuie sur un vaste ensemble de données internationales, nous visons 1) à décrire l’utilisation des anticonvulsivants pour traiter les troubles bipolaires du sujet âgé par rapport à leur utilisation pour traiter les troubles bipolaires du sujet jeune (< 50 ans) et 2) à explorer toute corrélation démographique/clinique.

**Méthodes:**

Une analyse secondaire des données internationales tirées du projet *Global Aging and Geriatric Experiments in Bipolar Disorder* (GAGE-BD) a été menée. L’objectif principal était de faire état de la prévalence de l’utilisation d’anticonvulsivants pour traiter les troubles bipolaires du sujet âgé chez les personnes de plus de 50 ans (âge moyen : 62,27 ans) et de l’anticonvulsivant le plus prescrit. Une analyse supplémentaire a permis d’examiner chaque corrélat démographique et clinique associé à l’utilisation d’anticonvulsivants. Des modèles linéaires mixtes généralisés ont été utilisés dans le cadre de cette analyse.

**Résultats:**

Sur les 2 691 participants atteints de trouble bipolaire pour lesquels des données sur la prescription d’anticonvulsivants étaient disponibles, 34,4% (n = 926) prenaient des anticonvulsivants au moment de l’étude. Les taux de prescription d’anticonvulsivants ne différaient pas de façon significative entre les groupes de troubles bipolaires du sujet âgé et le groupe de troubles bipolaires du sujet jeune (36,7% [n = 666] contre 29,7% [n = 260]). Les modèles de prescription d’anticonvulsivants pour les troubles bipolaires du sujet âgé et les troubles bipolaires du sujet jeune ne différaient pas de façon significative, le valproate étant l’anticonvulsivant le plus prescrit. Les personnes atteintes de troubles bipolaires du sujet âgé qui prenaient des anticonvulsivants ont pris moins de lithium et plus d’antidépresseur, ont présenté des cycles plus rapides, davantage de fluctuations de l’humeur et davantage de maladies cardiovasculaires concomitantes par rapport à celles qui n’en prenaient pas.

**Conclusion:**

L’utilisation d’anticonvulsivants était similaire pour traiter les troubles bipolaires du sujet âgé et ceux du sujet jeune. Un certain nombre de corrélats cliniques importants de l’utilisation d’anticonvulsivants ont été définis.

## Introduction

Bipolar disorder (BD) affects 0.5–1.0% of older adults,^1^ and with the rapidly aging population, this number of individuals with older age bipolar disorder (OABD) will only continue to grow. Of all mood disorders, BD has the largest negative impact on individual well-being, as well as the highest cost to healthcare systems.^2^ A wide domain of research has aimed to address which medications are best suited to manage BD. However, recommendations for OABD are limited, as most of the studies currently available have focused on younger adults.^3^

This leaves clinicians with minimal direction to prescribe medications for OABD.^4,5^ Lithium is often hailed as the gold standard treatment for BD, as determined in the Delphi survey of maintenance lithium treatment.^5–7^ However, concerns about potential renal complications have led clinicians to avoid recommending the use of lithium for the management of younger and older adults with BD.^5,6^ Consequently, there has been a significant increase in the use of anticonvulsants over the past three decades, making them the second most frequently prescribed class of medication after antidepressants.^4,7,8^ Numerous randomized controlled trials have demonstrated the efficacy of these medications in managing acute mania and bipolar depression.^8^ However, anticonvulsants have unique side effects and safety profiles, necessitating specific precautions for patient safety during both short-term and long-term use.^8,9^ While existing literature addresses anticonvulsant prescription guidelines for younger populations with BD, there is a noticeable gap in research on the use of anticonvulsants in OABD. This gap extends to the clinical correlates of anticonvulsant use in this population, highlighting the need for further investigation in this area.

Using the Global Aging & Geriatric Experiments in Bipolar Disorder Database (GAGE-BD) project dataset,^10^ we examined the prevalence of anticonvulsant use in OABD compared to younger adults with bipolar disorder (YABD, age <50). We also determined the most commonly prescribed anticonvulsants used, and sociodemographic and clinical correlates of anticonvulsant use that could provide helpful information to guide clinicians on anticonvulsant use in OABD.

## Methods

### Participants and Dataset

Participants were recruited for a large multisite cross-sectional international study for the GAGE-BD project.^10^ There were 32 contributing study cohorts and 17 sites for this dataset. Tables S1 and S2 describe the inclusion/exclusion criteria for each study. Of the 3,429 participants in the Wave 1 and 2 harmonized GAGE-BD dataset, we identified a total of 2,691 participants with BD and with anticonvulsant prescribing data. This sample was split into two categories: OABD (n = 1,815) and YABD (n = 876). The age cut off for OABD was determined to be ≥50 years old, as suggested by the ISBD Task Force due to the shortened life-span of BD.^11^ The methods were previously described by Sajatovic et al., 2015.^10,11^ All methods were performed in accordance with ethics guidelines at sites of contributing data and approved by relevant research ethics boards.

### Variables of Interest

The primary outcome of this secondary analysis was anticonvulsant use (yes/no), with secondary outcomes to report on prescription patterns between OABD and YABD, including the most prescribed anticonvulsant. Exploratory variables included any sociodemographic or clinical correlates such as age, sex, smoking status, number of hospitalizations, harmonized depression score, cognitive function (Mini-Mental State Examination),^12^ medical comorbidities, mania scores (Young Mania Rating Scale),^13^ among others. (Refer to Tables 1 and 2). Depression scores were measured by the Hamilton Depression Rating Scale (HAM-D),^14^ Montgomery-Asberg Depression Rating Scale (MADRS)^15^ or the Centre for Epidemiologic Studies Depression Scale (CES-D).^16^ This data was harmonized into one categorical depression variable with the following cut-offs: No depression (HAM-D ≤ 7; MADRS ≤6; CES-D ≤ 15), mild-moderate depression (HAM-D 8–23; MADRS 7–34; CES-D 16–27) and severe depression (HAM-D ≥ 24; MADRS ≥35; CES-D ≥ 28).^17^

**Table 1.** Demographic and Clinical Correlates of OABD in Anticonvulsant Users and Nonusers (n = 1815).

Correlates (n = 1815)	Anticonvulsant users (n = 666)	Anticonvulsant nonusers (n = 1149)	Statistics
**Age**	61.71 (SD = 8.33)	62.60 (SD = 8.77)	t(1) = 1.257, p = .209
**Sex (Female)**	59.0% (n = 393)	57.2% (n = 657)	t(1) = .049, p = .961
**Age of onset**	31.75 (SD = 14.72)	32.71 (SD = 15.39)	t(1) = 1.435, p = .152
**Education**	12.41 (SD = 4.55)	12.83 (SD = 3.93)	t(1) = .489, p = .625
**Lithium users**	27.2% (n = 180)	44.8% (n = 510)	**t(1)** **=** **11.275, p** **<** **.001**
**Antidepressant users**	46.9% (n = 292)	34.9% (n = 375)	**t(1) = 3.885, p** **<** **.001**
**Antipsychotic users**	51.5% (n = 342)	43.4% (n = 496)	t(1) = .737, p = .461
**Antipsychotic generation**			F(2) = 1.814, p = .164
−1^st^ generation	3.9% (n = 12)	5.9% (n = 27)	
−2^nd^ generation	90.5% (n = 275)	90.8% (n = 416)	
-Both	5.6% (n = 17)	3.3% (n = 15)	
**Previously hospitalized (yes)**	69.8% (n = 308)	69.2% (n = 467)	t(1) = .517, p = .605
**Number of hospitalizations**	3.29 (SD = 5.274)	2.67 (SD = 4.005)	t(1) = 1.835, p = .067
**Rapid cycling**			**F(2)** **=** **6.242, p** **=** **.002**
-No	77.3% (n = 228)	91.6% (n = 488)	
-Yes	20.3% (n = 60)	6.8% (n = 36)	
-Other	2.4% (n = 7)	1.7% (n = 9)	
**Number of affective episodes**	38.52 (SD = 201.70)	14.34 (SD = 40.17)	**t(1) = 2.278, p** **=** **.023**
**Cardiovascular comorbidity**	52.2% (n = 302)	43.2% (n = 441)	**t(1)** **=** **3.275, p** **=** **.001**
**Respiratory comorbidity**	35.3% (n = 169)	34.3% (n = 311)	t(1) = .005, p = .996
**Gastrointestinal comorbidity**	26.6% (n = 114)	22.4% (n = 182)	t(1) = 1.522, p = .128
**Hepatic/Pancreatic comorbidity**	8.7% (n = 34)	6.8% (n = 51)	t(1) = .918, p = .359
**Renal comorbidity**	7.1% (n = 29)	6.3% (n = 46)	t(1) = .137, p = .891
**Genito-urinary comorbidity**	21.5% (n = 73)	20.5% (n = 127)	t(1) = .980, p = .327
**Musculoskeletal comorbidity**	39.8% (n = 179)	41.1% (n = 337)	t(1) = .237, p = .813
**Endocrine comorbidity**	39.9% (n = 225)	33.8% (n = 336)	t(1) = 1.426, p = .154
**Smoking status**			F(3) = 1.326, p = .264
-Never	22.3% (n = 85)	18.4% (n = 140)	
-Current	31.7% (n = 121)	35.2% (n = 267)	
-Past	17.8% (n = 68)	12.3% (n = 93)	
-Unclear	28.3% (n = 108)	34.1% (n = 259)	
**Cognition: MMSE score**	27.05 (SD = 3.253)	27.59 (SD = 2.465)	t(1) = 1.492, p = .136
**Harmonized Depression**			F(2) = .114, p = .893
-No	62.0% (n = 330)	54.0% (n = 522)	
-Mild to moderate	33.6% (n = 179)	41.9% (n = 405)	
-Severe	4.3% (n = 23)	4.1% (n = 40)	
**Mania** (YMRS total)	4.78 (SD = 7.77)	8.0 (SD = 10.50)	t(1) = .219, p = .826

The bold values indicate the significant of p valuses < .05. OABD = older age bipolar disorder.

**Table 2.** Demographic and Clinical Correlates of YABD in Anticonvulsant Users and Nonusers (n = 876).

Correlates (n = 876)	Anticonvulsant users (n = 260)	Anticonvulsant nonusers (n = 616)	Statistics
**Age**	39.87 (SD = 7.17)	36.07 (SD = 8.81)	**t(1) = 2.128, p** **=** **.034**
**Sex (Female)**	62.7% (n = 163)	68.5% (n = 421)	t(1) = 1.501, p = .134
**Age of onset**	23.33 (SD = 9.60)	24.97 (SD = 9.51)	t(1) = .484, p = .628
**Education**	13.40 (SD = 3.91)	13.54 (SD = 3.22)	t(1) = .921, p = .357
**Lithium users**	30.8% (n = 80)	30.2% (n = 186)	**t(1) = 3.949, p** **<** **.001**
**Antidepressant users**	45.6% (n = 118)	37.5% (n = 230)	t(1) = .133, p = .894
**Antipsychotic users**	57.5% (n = 149)	44.2% (n = 272)	t(1) = .599, p = .549
**Antipsychotic generation**			F(2) = .825, p = .439
**−**1^st^ generation	4.4% (n = 6)	4.8% (n = 12)	
−2^nd^ generation	94.8% (n = 128)	94.0% (n = 237)	
-Both	0.7% (n = 1)	1.2% (n = 3)	
**Previously hospitalized (yes)**	74.4% (n = 163)	60.7% (n = 334)	t(1) = 1.084, p = .279
**Number of hospitalizations**	3.82 (6.07)	2.17 (4.12)	**t(1) = 2.047, p** **=** **.041**
**Rapid cycling**			F(2) = .889, p = .412
-No	39.3% (n = 22)	64.4% (n = 177)	
-Yes	58.9% (n = 33)	33.5% (n = 92)	
-Other	1.8% (n = 1)	2.2% (n = 6)	
**Number of affective episodes**	27.85 (SD = 82.44)	20.77 (SD = 57.30)	t(1) = .306, p = .760
**Cardiovascular comorbidity**	21.8% (n = 38)	7.7% (n = 35)	t(1) = 1.486, p = .138
**Respiratory comorbidity**	35.2% (n = 44)	14.6% (n = 61)	t(1) = 1.235, p = .217
**Gastrointestinal comorbidity**	13.4% (n = 18)	5.9% (n = 25)	t(1) = .342, p = .732
**Hepatic/Pancreatic comorbidity**	2.4% (n = 3)	0.7% (n = 3)	t(1) = .769, p = .442
**Renal comorbidity**	2.2% (n = 3)	0.7% (n = 3)	t(1) = .932, p = .352
**Genito-urinary comorbidity**	2.5% (n = 2)	1.2% (n = 4)	t(1) = .449, p = .654
**Musculoskeletal comorbidity**	30.7% (n = 39)	13.9% (n = 58)	t(1) = .505, p = .614
**Endocrine comorbidity**	14.6% (n = 24)	9.6% (n = 42)	t(1) = .370, p = .712
**Smoking status**			F(3) = 1.762, p = .153
-Never	12.8% (n = 24)	25.1% (n = 124)	
-Current	50.3% (n = 94)	46.7% (n = 231)	
-Past	3.2% (n = 6)	7.5% (n = 37)	
-Unclear	33.7% (n = 63)	20.8% (n = 103)	
**Cognition: MMSE score**	24.33 (4.62)^a^	24.75 (3.20)^b^	t(1) = .731, p = .498
**Depression**			F(2) = .154, p = .857
-No	44.8% (n = 73)	33.2% (n = 151)	
-Mild to moderate	49.7% (n = 81)	62.4% (n = 284)	
-Severe	5.5% (n = 9)	4.4% (n = 20)	
**Mania (YMRS total)**	7.29 (SD = 8.78)	6.08 (SD = 6.56)	t(1) = .199, p = .842

^a^n = 3; ^b^n = 4.

The bold values indicate the significant p values < .05. YABD=younger age bipolar disorder; MMSE=Mini-Mental State Examination.

### Statistical Analysis

Statistical analyses were conducted using IBM SPSS Statistics (Version 29). Demographic and clinical characteristics were described using frequencies, percentages, means and standard deviations. Independent t-tests and chi-square tests, and generalized linear mixed models (GLMM) were used to explore relationships between OABD vs YABD for primary, secondary and exploratory outcomes. GLMM using a binomial probability distribution with a logit link function and random effect of study cohort were used to control for study cohorts.

## Results

### Demographics and Clinical Characteristics

Table 3 summarizes the demographic and clinical characteristics of the sample. The mean age for OABD and YABD was 62.27 (SD = 8.62) and 37.20 (SD = 8.53), respectively. There was a higher proportion of females with YABD (66.7%) compared to OABD (57.9%), χ2(1) = 19.57, p < .001. The mean age of onset was higher in participants with OABD (32.37, SD = 15.16) compared to YABD (24.47, SD = 9.56), p < .001, 95% CI [-8.95, −6.86].

**Table 3.** Demographic Characteristics of Participants with Bipolar Disorder from the GAGE-BD Dataset (n = 2691).

	OABD (≥50 years old) (n = 1815)	YABD (<50 years old) (n = 876)
**Age** (years)	62.3 (SD = 8.62)	37.2 (SD = 8.53)
Mean (SD)		
**Sex** (Female)	57.9% (n = 1050)	66.7% (n = 584)
**Diagnosis** (BD Sub-Type)	BD 1: 76.7% (n = 1381)	BD 1: 73.0% (n = 634)
	BD 2: 20.4% (n = 368)	BD 2: 23.6% (n = 205)
	Unknown: 2.9% (n = 52)	Unknown: 3.3% (n = 29)
**Age of onset**	32.4 (SD = 15.16)	24.5 (SD = 9.56)
Mean (SD)		
**Education in years**	12.7 (SD = 4.15)	13.5 (SD = 3.43)
Mean (SD)		
**Occupation** (working)	23.1% (n = 257)	37.0% (n = 179)
**Relationship status**		
-In relationship	40.2% (n = 594)	35.2% (n = 173)
-Single	57.9% (n = 856)	64.4% (n = 316)
-Unknown	1.9% (n = 28)	0.4% (n = 2)
**Hospitalized for psychiatric condition (yes)**	69.4% (n = 775)	64.6% (n = 497)

OABD=older age bipolar disorder; YABD=younger age bipolar disorder; GAGE-BD=Global Aging and Geriatric Experiments in Bipolar Disorder.

### Anticonvulsant use in OABD vs. YABD

Anticonvulsant use was 36.7% in OABD and 29.7% in YABD, t(1) = 1.40, p = .161 (Table 4). The number of anticonvulsants used was similar for OABD and YABD, with most participants using only one anticonvulsant.

**Table 4.** Anticonvulsant users in OABD vs YABD (n = 2,691).

	OABD (≥50 years old) (n = 1815)	YABD (<50 years old) (n = 876)	Statistics
**Anticonvulsant use; % (n)**	36.7% (n = 666)	29.7% (n = 260)	t(1) = 1.40, p = .161
**Number of anticonvulsants; % (n)**	0: 63.3% (n = 1149)	0: 70.3% (n = 616)	F(4) = .143, p = .966
	1: 33.2% (n = 603)	1: 26.8% (n = 235)	
	2: 3.3% (n = 60)	2: 2.6% (n = 23)	
	3: 0.2% (n = 3)	3: 0.1% (n = 1)	
	4: 0% (n = 0)	4: 0.1% (n = 1)	

OABD=older age bipolar disorder; YABD=younger age bipolar disorder.

### Prescription Patterns of Anticonvulsant use in OABD vs YABD

Table 5 describes the different anticonvulsants prescribed for OABD and YABD. Prescription patterns did not differ significantly between OABD and YABD. Valproate (VPA) was the most commonly used anticonvulsant (OABD: VPA comprised 46.4% of all anticonvulsant use in OABD; YABD: 41.2%) followed by lamotrigine (OABD: 34.5%; YABD: 35.4%). Although gabapentin had a p < 0.05, our significance was set to p = 0.005 due to Bonferroni correction and therefore gabapentin use was not significantly different between OABD and YABD groups.

**Table 5.** Prescribing Patterns Amongst OABD and YABD Anticonvulsant Users (n = 926).

Name of anticonvulsant	OABD (≥50 years) (n = 666)	YABD (<50 years) (n = 260)	Statistics
***Carbamazepine***	13.1% (n = 87)	13.5% (n = 35)	χ2(1) = .026, p = .872
***Oxcarbazepine***	4.1% (n = 27)	5.0% (n = 13)	χ2(1) = .405, p = .525
***Gabapentin***	5.3% (n = 35)	8.8% (n = 23)	χ2(1) = 4.107, p = .043
***Lamotrigine***	34.5% (n = 230)	35.4% (n = 92)	χ2(1) = .060, p = .807
***Pregabalin***	1.4% (n = 9)	0.8% (n = 2)	Fisher's Exact Test, p = .737
***Primidone***	0.2% (n = 1)	0% (n = 0)	Fisher's Exact Test, p = 1.0
***Topiramate***	4.2% (n = 28)	5.8% (n = 15)	χ2(1) = 1.034, p = .309
***Valproate***	46.4% (n = 309)	41.2% (n = 107)	χ2(1) = 2.077, p = .150
***Levetiracetam***	0% (n = 0)	0.4% (n = 1)	Fisher's Exact Test, p = .281
***Other***	1.8% (n = 12)	0% (n = 0)	Fisher's Exact Test, p = .024

*Statistical sig is when p *<* *0.05/10* *<* *0.005. YABD=*younger age bipolar disorder.

### Correlates of Anticonvulsant use

Tables 1 and 2 summarize the demographic and clinical correlates of anticonvulsant use in OABD (Table 1) and YABD (Table 2). Of the 1,815 older adults who have BD, n = 666 were anticonvulsant users and n = 1,149 were nonusers.

In OABD anticonvulsant users (compared to nonusers) (Table 1), lithium use was less common (27.2% vs. 44.8%, t(1) = 11.275, p < .001), while antidepressant use was more common (46.9% vs. 34.9%, t(1) = −3.885, p < .001). Cardiovascular comorbidities were more common in anticonvulsant users compared to nonusers (52.2% vs. 43.2%, t(1) = 3.275, p = .001). Rapid cycling was also more common in anticonvulsant users (20.3% vs. 6.8%, F(2) = 6.242, p = .002), and the number of lifetime mood episodes was higher (mean = 38.52 vs. 14.34). The age, proportion of females, age of onset, education level, antipsychotic use, number of hospitalizations, other comorbidities, cognition, as well as depression and mania scores were not significantly different in anticonvulsant users and nonusers.

In YABD (Table 2), the mean age for anticonvulsant users and nonusers was 39.87 (SD = 7.17) and 36.07 (SD = 8.81), respectively, t(1) = −2.128, p = .034. Lithium use was more common in anticonvulsant users compared to nonusers (30.8% vs. 30.2%, t(1) = 3.949, p < .001), and number of hospitalizations was also higher in anticonvulsant users compared to nonusers (3.82 vs 2.17, t(1) = −2.047, p = .041). The proportion of females, age of onset, education level, antidepressant use, antipsychotic use, comorbidities, cognition, depression and mania scores were not significantly different in anticonvulsant users and nonusers.

## Discussion

This paper leveraged the large international GAGE-BD dataset to 1) describe the use of anticonvulsants in OABD compared to YABD, and 2) explore any demographic/clinical correlates. We had anticonvulsant prescribing data on 2,691 individuals with BD, including OABD (n = 1,815) and YABD (n = 876). We also identified important correlates of anticonvulsant use in OABD.

In this analysis, anticonvulsant use in OABD (36.7%) was not statistically different compared to YABD (29.7%). Clinicians may be following similar prescribing patterns for both older and younger adults, possibly due to the limited availability of clinical trials and specific guidelines tailored to older populations.^18^ Although the similar use of anticonvulsants in OABD and YABD may be due to anticonvulsants being perceived as safer than alternative medications in OABD, e.g., VPA is safer than lithium regarding kidney risk.^18,19^ The previous literature cites similar potential reasons for the (relatively) more common anticonvulsant use in OABD.^18,19^ Possible additional explanations for this could be gleaned from examining the concurrent prescribing patterns of other psychotropic medications in OABD anticonvulsant users (e.g., antipsychotic use – a medication that requires less frequent blood monitoring).

The type of anticonvulsant prescribed was similar amongst anticonvulsant users regardless of age (OABD vs YABD), with VPA (46.4%) being the most prescribed anticonvulsant, followed by lamotrigine (34.5%) and carbamazepine (13.1%). In our current era this level of use of carbamazepine may seem high in older adults given its hematological concerns and propensity towards drug–drug interactions. However, perhaps this speaks to carbamazepine's efficacy^20^ and clinicians’ wish to use it despite its adverse effect profile. This suggests that the prescribers may not perceive differences between OABD and YABD in terms of anticonvulsants’ efficacy and safety. Since there are relatively few randomized clinical trials on OABD,^21^ most research suggests similar prescribing patterns to YABD and this can be seen in the current data. Future studies could assess a few interesting hypotheses: (1) whether clinicians find certain anticonvulsants to be more efficacious than the more commonly prescribed medications (antipsychotics) and (2) whether that explains higher anticonvulsant use despite many clinicians’ perceptions that antipsychotics are safer (require less monitoring, less drug–drug interactions).

There are some interesting clinical correlates of anticonvulsant use in OABD. Lithium use was significantly lower in anticonvulsant users compared to nonusers. It is possible that physicians perceive lithium use as riskier in many OABD patients^19^ and opt to prescribe anticonvulsants in those patients. Alternatively, the sample collected could reflect a lithium treatment resistant group. In contrast, for YABD, anticonvulsant use was associated with more lithium use, which likely illustrates the hesitancy to prescribe lithium to older adults. The results also demonstrate that anticonvulsant users (compared to nonusers) more commonly use antidepressants, suggesting that anticonvulsants are used in more atypical forms of BD (not the typical familial classical lithium-responsive BD^22^), or that multiple medication classes are needed. Anticonvulsant users also had more rapid cycling and a higher amount of mood episodes, which suggests that anticonvulsants are again used in the more atypical forms of BD. Future hypotheses that could be tested include: (1) whether clinicians tend to switch patients from lithium to anticonvulsants around age 60, and (2) prospectively compare the efficacy of anticonvulsants vs lithium vs antipsychotics in OABD, including subgroup analyses of atypical forms of BD.

Cardiovascular comorbidities were more prevalent in anticonvulsant users (compared to nonusers), which is consistent with the literature that suggests individuals with BD have an elevated risk for cardiovascular mortality.^23,24^ In addition, anticonvulsant use has also been associated with metabolic changes (in cytochrome P450 enzyme) that may increase cardiovascular risk.^25,26^ This suggests a few possibilities, 1) physicians perceive anticonvulsant use to be safer for OABD patients with cardiovascular comorbidities, or 2) anticonvulsant use increases cardiovascular risk. However, due to the cross-sectional nature of this study, causality cannot be inferred, and future research is needed.

## Strengths and Limitations

There are many strengths of this study, such as its large sample size (n = 2,691), and the use of various international sites, which increases the external validity of the findings. An important limitation includes the cross-sectional nature of this study, which has difficulty establishing temporality or causality.^27^ The data collected was from a sample with a mean age of 62, which is not necessarily generalizable to people in their mid-70s or older. In addition, the data was collected over a long period of time (∼20 years) and the exact dates of data collection for each study were not available, so we could not examine the impact of pharmaceutical marketing on prescription patterns. Due to the heterogeneity of OABD, including those with both early-onset and late-onset BD can be seen as a limitation since we did not separate these subgroups for our analysis.

Although we have used the GLMM for our primary analysis, we used a chi-square to compare younger and older adults’ type of anticonvulsant use (e.g., VPA, lamotrigine, etc.). Future studies can further examine types of anticonvulsant use using GLMM and 95% confidence intervals. In addition, we did not have information about efficacy, adverse effects, or medication adherence, which could be assessed in future longitudinal studies.

## Conclusions

Our data suggest anticonvulsants are widely used in OABD, with VPA as the most common anticonvulsant for clinicians treating OABD. Anticonvulsant use was similar for OABD and YABD, and prescription patterns of individual anticonvulsants remained similar regardless of age. The clinical correlates of anticonvulsant use in OABD include less lithium use, more antidepressant use, more rapid cycling, higher number of mood episodes and more cardiovascular comorbidities. Future longitudinal studies could help us understand the relationship between anticonvulsant use and the correlates identified.

## Data Access

The data are available from the corresponding author on reasonable request.

## Supplemental Material

sj-docx-1-cpa-10.1177_07067437251372190 - Supplemental material for Anticonvulsant Use in Older Age Bipolar Disorder in a Global Sample from the Global Aging and Geriatric Experiments in Bipolar Disorder Project: Utilisation d’anticonvulsivants pour le traitement des troubles bipolaires du sujet âgé auprès d’un échantillon mondial provenant du projet GAGE-BD Supplemental material, sj-docx-1-cpa-10.1177_07067437251372190 for Anticonvulsant Use in Older Age Bipolar Disorder in a Global Sample from the Global Aging and Geriatric Experiments in Bipolar Disorder Project: Utilisation d’anticonvulsivants pour le traitement des troubles bipolaires du sujet âgé auprès d’un échantillon mondial provenant du projet GAGE-BD by Katie C. Bodenstein, Myriam Lesage, Paola Lavin, Sigfried Schouws, Melis Orhan, Alexandra Beunders, Osvaldo P. Almeida, Kursat Altinbas, Vicent Balanzá-Martínez, Izabela G. Barbosa, Hilary P. Blumberg, Farren B.S. Briggs, Cynthia V. Calkin, Orestes V. Forlenza, Brent Forester, Ariel G. Gildengers, Benno C.M. Haarman, Tomas Hajek, Beny Lafer, Paula Nune, Benoit Mulsant, Andrew T. Olagunju, Regan E. Patrick, Joquim Radua, Kaylee Sarna, Christian Simhandl, Jair C. Soares, Ashley N. Sutherland, Nicole Fiorelli, Antonio L. Teixeira, Shangying Tsai, Eduard Vieta, Joy Yala, Lisa Eyler, Annemiek Dols, Martha Sajatovic and Soham Rej in The Canadian Journal of Psychiatry
